# Book review: Researching classroom discourse

**DOI:** 10.3389/fpsyg.2022.985873

**Published:** 2022-09-09

**Authors:** Yanfang Hou, Jun Zhang

**Affiliations:** ^1^School of Foreign Studies, Yangtze University, Jingzhou, China; ^2^School of Foreign Languages, Wuhan University of Science and Technology, Wuhan, China

**Keywords:** classroom discourse, research guide, novice researchers, research methods, research overview

The book to be introduced is Christopher J. Jenk's latest works, Researching Classroom Discourse, published by Routledge in 2021 (Jenks, 2020).

In recent years, there is a growing interest in classroom discourse research from different perspectives, including cognitive interaction theory, sociocultural theory, complexity theory, language socialization theory, post-structuralism theory, etc. However, despite this increased interest in the past few decades, most books focus on theoretical or methodological issues or report detailed research findings while less has been done to address the writing and research needs of novice researchers and university students. And there has not been a book that attempts to provide an accessible guide and overview on how to conduct such research on classroom discourse. The book under review fills the gap and intends to help novice researchers and readers preparing to be teachers or teacher trainers conduct classroom discourse research by providing an easy-to-understand overview and step-by-step guidance of the research process.

The book is the result of the author's years of teaching and supervising in a variety of countries. Offering a comprehensive overview of the research process, this book aims to help readers put the theoretical and methodological issues of classroom discourse analysis into practice.

The book consists of eight chapters grouped into three parts around three stages of doing research: planning (Part I), analyzing (Part II), and understanding and reporting (Part III). Each chapter covers a range of theoretical, thematic, and analytic issues by providing helpful examples, data, reflective questions, and sources for further reading.

Part I (Chapters 1–2) introduces the key issues of research planning. By reviewing and synthesizing relevant studies, Chapter 1 discusses definitions of classroom research and classroom discourse analysis and highlights the significance, motivations, and justifications for doing classroom discourse research to help readers gain a general understanding of classroom discourse research. Instead of a lengthy and complicated explanation of the theoretical concepts, this chapter attempts to guide readers to reflect on general methodological considerations of classroom discourse research. It identifies five methodological issues (data collection, data presentation, type of analysis, level of analysis, and role of context) to be addressed throughout the following chapters. While the first chapter informs readers of the fundamental concepts in classroom discourse research, Chapter 2 elaborates on important considerations in carrying out the research. After offering a detailed introduction to five logistical aspects (access, time, ethics, technology, and empirical issues) involved in doing research, the chapter specifies principles, tools, and methods of collecting and transcribing classroom discourse data.

Part II (Chapters 3–6) provides a comprehensive introduction to four major approaches to classroom discourse which shape the types and levels of analysis. Chapter 3 sketches out a concise but informative introductory account of what conversation analysis (CA) entails and what its benefits for classroom discourse research are. Four key classroom discourse issues in CA are reviewed in the chapter, namely, turn taking, turn design, repair, and interactional competence. To help readers gain an in-depth understanding of the nature of CA, the author emphasizes that CA engages in micro-level research with a focus on spoken communication, and that CA studies should rely on naturally occurring data. Therefore, he suggests that researchers engage in unmotivated looking, collecting, and analyzing data without the guidance of predefined empirical objectives to present the emic nature of classroom discourse.

Chapter 4 is mainly dedicated to providing an overview of discourse analysis (DA), an interdisciplinary approach to uncovering how discourse-level features and phenomena relate to teaching and learning. The chapter begins with introductions to four discourse phenomena that are commonly investigated: triadic dialogue [initiation– response–feedback (IRF)/initiation–response–evaluation (IRE) sequence], floor management, teacher questions, and discourse markers. It is suggested that a successful DA investigation should at least accomplish two analytic objectives: describing and contextualizing. Describing the features of classroom discourse is helpful to get the whole picture of the discourse, while contextualizing helps to effectively identify DA's pedagogical function.

Given the complex and dynamic nature of classroom discourses (Walsh, 2006), the analysis may entail multiple levels from the micro to the macro. Critical discourse analysis (CDA) meets this demand and is the theme of Chapter 5. It provides a practical overview of CDA by illustrating how classroom discourse is embedded in the larger social context and is inevitably connected to social issues, including power relations, language ideologies, neoliberalism, and racism. The chapter proposes that classroom discourse researchers establish connections between what happens in the act of teaching and learning with issues outside the classrooms.

Chapter 6 is dedicated to providing an in-depth introduction to narrative analysis (NA), an approach used to investigate reflection data concerning teaching and learning. Four areas that are commonly investigated in narrative studies, including identities, teacher cognition, reflective practices, and learner diaries, are presented. By so doing, the author provides insightful ideas and thus a sufficient foundation for readers to conduct narrative analysis and combine classroom processes with learner/teacher psychology as well as social contexts.

Part III (Chapters 7–8) is dedicated to providing guidance on how to understand and report the classroom discourse research.

Chapter 7 introduces how an ethnographic approach can be used in classroom discourse research and how this approach helps to uncover the multifaceted nature of classroom discourse. In particular, two types of ethnographic approaches are discussed: ethnography of communication and autoethnography. While ethnography of communication helps to investigate how members of speech communities learn, demonstrate, and co-construct communicative competence, autoethnography examines the experiences and feelings of the researcher in a classroom research context.

Chapter 8 centers on how to report and write up the observations made in classroom discourse research. In a linear process from pre-writing to drafting, the author shows readers, in a step-by-step manner, how an effective classroom discourse research report can be drafted. More specifically, the author explicates what principles should be followed in classroom discourse research, how to describe the methodology, and how to effectively present the data.

One of the most significant strengths of this book lies in its focus on practice. The aim of it is appealing: to provide novice researchers with accessible guidance on how to carry out classroom research so that they can put the theoretical and methodological issues of classroom discourse analysis into practice. The theories covered in this book are explored by providing comprehensive and up-to-date overviews and the methodologies are discussed in depth to offer accessible and easy-to-follow guidance and direction for novice researchers to conduct their own research. The knowledge provided by the book, combined with the subsequent actions that the book suggests the readers take is helpful for researchers and teaching practitioners to acquire a nuanced understanding of classroom discourse processes, and thus build strong connections between research and pedagogy (Llinares, 2015). Each chapter is discussed in an application-oriented manner and organized largely around reflective questions and reading lists that are thought-provoking, encouraging readers to reflect on their empirical studies and to extend their knowledge of classroom learning. The guidance and suggestions offered by this book are practical and easy to follow, and thus lay a solid foundation for novice researchers.

Another advantage of this book lies in its interdisciplinary perspective. The book is not limited to guiding students to investigate the surface linguistic features and cognitive processes in classrooms, but also looks at the social and cultural dimensions of classroom discourse, analyzing it from such perspectives as sociology, psychology, anthropology, etc. What impresses us most are the inspiring and cutting-edge thoughts and notions advocated in the book. For example, it highlights the necessity to put English teaching research in the context of contemporary global politics, economy, and culture, hence guiding readers to understand the complexity of classroom discourse by connecting both micro and macro levels. In addition to conventional research methodologies, some more recent approaches to analyzing classroom discourse, such as ethnography and autoethnography, are also introduced.

The structure of the book is reader-friendly, since it is well-organized following the research process from research planning, and analysis to reporting. Each chapter is attached with recommended publications, which is helpful for novice researchers to better understand the issues discussed and carry out their own research projects. As a guidebook for novice researchers, theories and methodologies are presented step by step without cumbersome explanations, making it highly readable and easy to follow. In particular, the “Key Terms, Constructs and People” section at the end of each chapter provides readers with a clear guide to better understand the core ideas and helps readers access some of the most important academic sources for their present and further studies.

Despite all these strengths, readers may find the book more comprehensive and illuminating if it provides a more detailed introduction to some quantitative methods (such as the corpus-based method) which are only mentioned without in-depth discussion in this book. Moreover, more studies concerning multimodal factors in classroom discourse in such contexts as synchronic online communication and synchronous computer-based communication could have been included.

All in all, this guidebook is of great value to novice researchers, postgraduates, and those with an interest in classroom discourse research.

## Author contributions

The article is coauthored by YH and JZ. Both authors first read the book simultaneously and then discussed their gains from the book. After reaching the agreement on the merits of the book, YH wrote the first draft of the review, with JZ giving constructive suggestions and making some revisions on the subsequent drafts. Therefore, the review is the result of their collective work based on their shared understanding of the book. Both authors contributed to the article and approved the submitted version.

## Funding

This work was supported by Hubei Provincial Philosophical and Social Science Research Project, China [Grant Number 19ZD021]; the Institute of Science and Technology Development, Yangtze University, China [Grant Number 2018csz07]; and the Philosophical and Social Science Research Project of Department of Education of Hubei Province [Grant Number 21Q024].

## Conflict of interest

The authors declare that the research was conducted in the absence of any commercial or financial relationships that could be construed as a potential conflict of interest.

## Publisher's note

All claims expressed in this article are solely those of the authors and do not necessarily represent those of their affiliated organizations, or those of the publisher, the editors and the reviewers. Any product that may be evaluated in this article, or claim that may be made by its manufacturer, is not guaranteed or endorsed by the publisher.
